# Zinc Transporter 8 and MAP3865c Homologous Epitopes are Recognized at T1D Onset in Sardinian Children

**DOI:** 10.1371/journal.pone.0063371

**Published:** 2013-05-17

**Authors:** Speranza Masala, Maria Antonietta Zedda, Davide Cossu, Carlo Ripoli, Mario Palermo, Leonardo A. Sechi

**Affiliations:** 1 Università degli Studi di Sassari, Dipartimento di Scienze Biomediche, Sezione di Microbiologia e Virologia, Sassari, Sardinia, Italy; 2 Clinica Pediatrica Macciotta, Cagliari, Sardinia, Italy; 3 Department of Medicine, Endocrinology Unit, Azienda Ospedaliero Universitaria (AOU), Sassari, Sardinia, Italy; Institut national de la santé et de la recherche médicale (INSERM), France

## Abstract

Our group has recently demonstrated that *Mycobacterium avium* subspecies *paratuberculosis* (MAP) infection significantly associates with T1D in Sardinian adult patients. Due to the potential role played by MAP in T1D pathogenesis, it is relevant to better characterize the prevalence of anti-MAP antibodies (Abs) in the Sardinian population, studying newly diagnosed T1D children. Therefore, we investigated the seroreactivity against epitopes derived from the ZnT8 autoantigen involved in children at T1D onset and their homologous sequences of the MAP3865c protein. Moreover, sera from all individuals were tested for the presence of Abs against: the corresponding ZnT8 C-terminal region, the MAP specific protein MptD, the T1D autoantigen GAD65 and the T1D unrelated Acetylcholine Receptor. The novel MAP3865c_281–287_ epitope emerges here as the major C-terminal epitope recognized. Intriguingly ZnT8_186–194_ immunodominant peptide was cross-reactive with the homologous sequences MAP3865c_133–141_, strengthening the hypothesis that MAP could be an environmental trigger of T1D through a molecular mimicry mechanism. All eight epitopes were recognized by circulating Abs in T1D children in comparison to healthy controls, suggesting that these Abs could be biomarkers of T1D. It would be relevant to investigate larger cohorts of children, followed over time, to elucidate whether Ab titers against these MAP/Znt8 epitopes wane after diagnosis.

## Introduction

Type 1 Diabetes (T1D) is a T cell-mediated autoimmune disease resulting from the destruction of insulin-secreting pancreatic β cells. Although it is established that this autoimmune disease stems from a combination of genetic predisposition and environmental factors, the latter remain elusive [1]. During the period preceding T1D clinical onset, autoantibodies (aAbs) directed to islets antigens such as insulin, glutamic acid decarboxylase (GAD65), insulinoma associated protein-2 and zinc transporter 8 (Znt8) may be detectable for months up to years before disease onset [2], and progressively wane after diagnosis [3]. Wenzlau *et al*. reported that 60–80% of recent-onset T1D harbor antibodies against Znt8 C terminal domain [4].

Our recent studies suggested that *Mycobacterium avium* subspecies *paratuberculosis* (MAP) infection significantly associates with T1D in Sardinian population [5]. We reported that anti-MAP and anti-ZnT8 antibodies (Abs) targeting homologous membrane-spanning sequences are cross-reactive and capable of eliciting strong immune responses in T1D adult patients [6], opening the possibility of a molecular mimicry mechanism precipitating disease.

Sardinia is one of the regions with the highest incidence of T1D and multiple sclerosis worldwide, displaying a much higher T1D prevalence compared to other Mediterranean populations [7]. Several factors, such as genetic isolation, have likely contributed to the fixing of predisposing alleles. MAP infection is estimated to affect approximately 60% of Sardinian herds [6], and this mycobacterium can be found in pasteurized milk products and may be asymptomatically transmitted to humans [8]. Due to the potential role played by MAP in T1D pathogenesis, it is relevant to better characterize the prevalence of anti-MAP Abs in the Sardinian population. To this end, we investigated the seroreactivity against the previously identified ZnT8/MAP epitopes, in children at T1D-onset and compared to healthy controls (HCs) [6]. Moreover, sera from all individuals were tested for the presence of Abs against 4 newly identified putatively relevant C-terminal MAP3865c epitopes (MAP3865c_246–252_, MAP3865c_256–262_, MAP3865c_261–267_ and MAP3865c_281–287_), the corresponding ZnT8 C-terminal region, the MAP specific protein MptD, the T1D autoantigen GAD65 and the T1D unrelated Acetilcoline Receptor (ACHR).

## Materials and Methods

### Subjects

Sardinian new-onset T1D children (n = 29; 15 boys, 14 girls; mean age 8.6±4 years; diabetes duration 4.3 days [0–25]); diagnosed according to the American Diabetes Association criteria [9]; and Sardinian HCs (n = 30; 14 boys, 21 girls; mean age 8±3) were enrolled in Cagliari and Sassari. Patients’ details are provided in Table S1. Serum samples were processed as previously described [6].

### Ethical Statement

Blood samples were collected after obtaining informed written consents from the guardians of all subjects. The study protocols were approved by the ethics committee of the University of Sassari and Cagliari, Sardinia, Italy.

### Peptides

Peptides MAP3865c_125–133_ (MIAVALAGL) and MAP3865c_133–141_ (LAANFVVAL) along with their respective homologous peptides ZnT8_178–186_ (MIIVSSCAV), ZnT8_186–194_ (VAANIVLTV) MAP3865c_246–252_ (LSPGKDM), MAP3865c_256–262_ (HLISTGD), MAP3865c_261–267_ (GDSARVL) and MAP3865c_281–287_ (HATVQID) were synthesized at >85% purity (GL Biochem).

### ELISA

Indirect enzyme-linked immunosorbent assay (ELISA) to detect Abs specific for MAP3865c peptides and MptD protein were performed as described elsewhere [6]. The cut-off for positivity was calculated by ROC analysis, setting specificity at 93.3% (i.e., Ab+ HCs ≤6.7%). The percent fraction of Ab+ sera, Area Under ROC Curve(AUC), and *p* values after Fisher exact test are indicated. Results were normalized to a strongly positive control serum included in all experiments, the reactivity of which was set at 10.000 arbitrary units (AU)/ml. The statistical significance was determined using Graphpad Prism 6.0 software.

ElisaRSR™ ZnT8 Ab™ (RSR Limited, United Kingdom) kit for the quantitative determination of autoantibodies (aAbs) to the ZnT8 C-terminal region (Znt8_275–369_) in serum was carried out according to the manufacturer’s instructions.

The concentration of both human GAD65 and human Acetylcholine Receptor (ACHR) Abs in serum samples from T1D patients and matched HCs was determined using two different ELISA kit (MBS, The Netherlands) following the manufacturer’s instructions.

## Results

**Figure 1:**
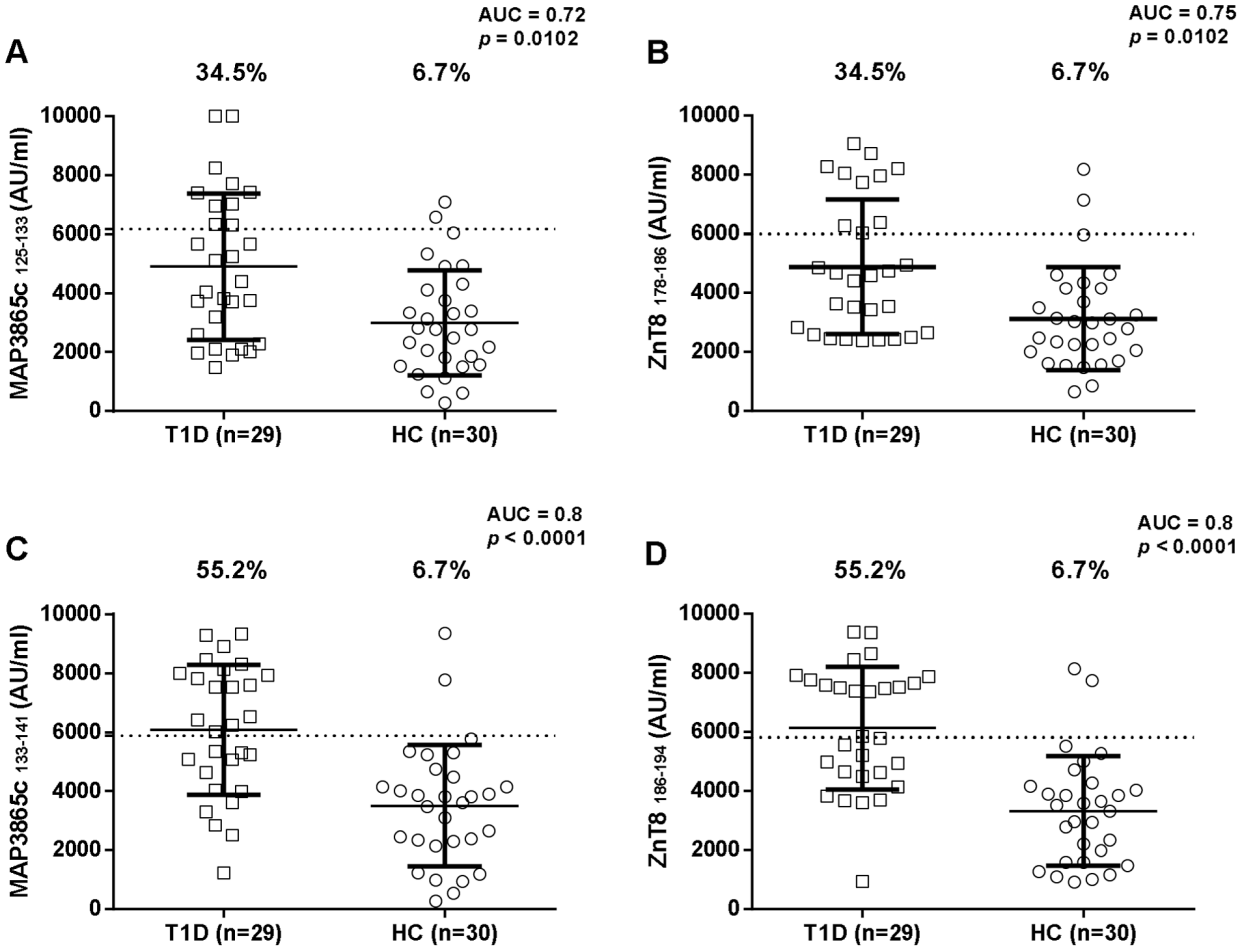
Prevalence of Abs against homologous ZnT8 and MAP3865c transmembrane epitopes in Type 1 Diabetes (T1D), and healthy controls (HCs) Sardinian children. Sera were tested for their reactivity against plate-coated with MAP3865c_125–133_(A) and its homologous ZnT8_178–186_ (B); and with MAP3865c_133–141_ (C) and its homologous ZnT8_186–194_ (D). The dotted line lines indicate the cut-off for positivity used in each assay, as calculated by ROC analysis. The percent fraction of Ab+ sera is indicated on top of each distribution, while bars indicate the corresponding median±interquartile range. AUC and *p* values are given in the top right corner. Figure shows representative experiments out of three performed.

**Figure 2:**
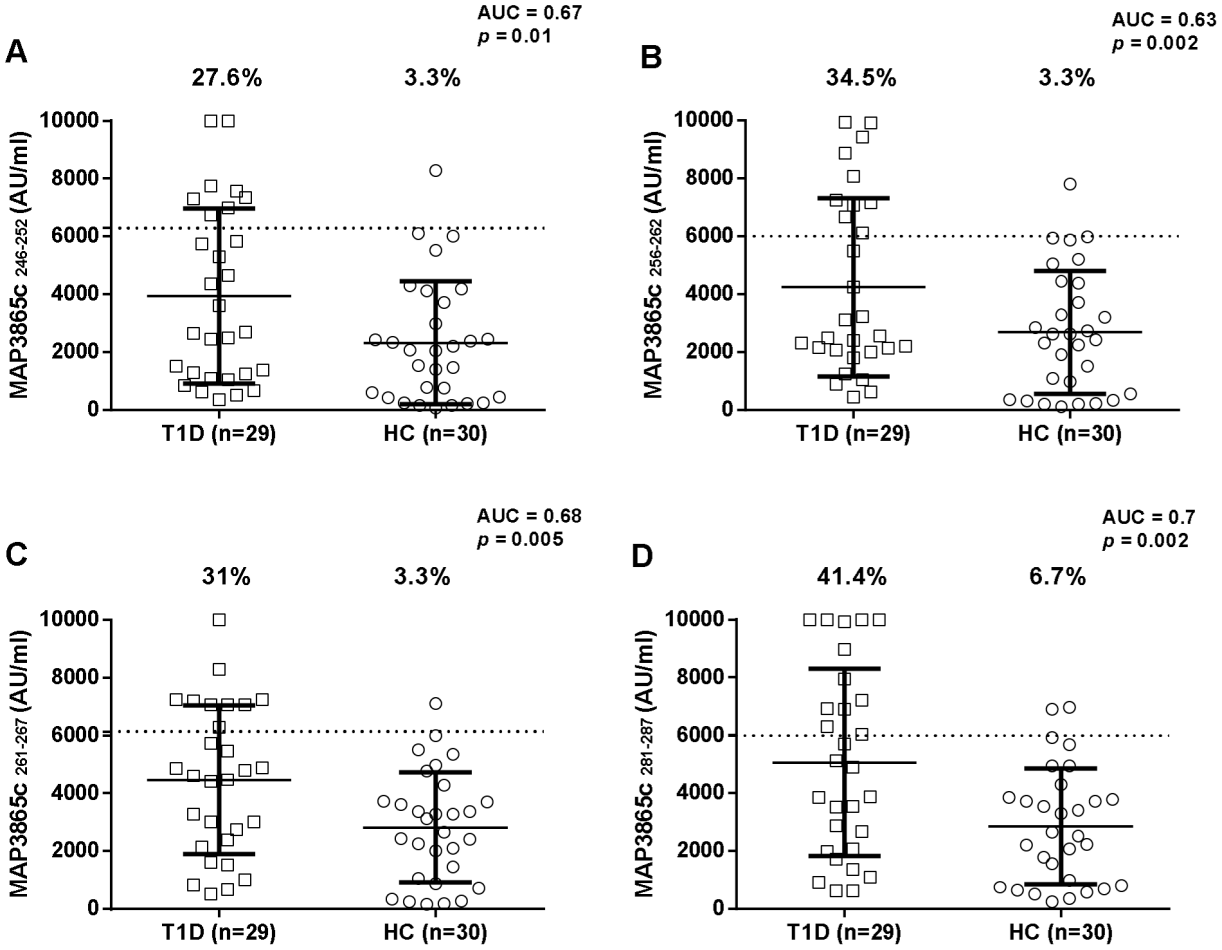
Prevalence of Abs against MAP3865c epitopes falling into the region of homology comprising the polymorphic Znt8 325^th^ residue in 29 Type 1 Diabetes (T1D) and 30 healthy controls (HCs) Sardinian children. Sera were tested for their reactivity against plate-coated with MAP3865c_246–252_ (A) MAP3865c_256–262_ (B) MAP3865c_261–267_ (C) and MAP3865c_281–287_ (D) peptides. Data representation is the same as in Fig. 1.

Abs responses against eight MAP/Znt8 peptides, four falling into the transmembrane region previously described [6], and four homologues to the human C-terminal Znt8 immunogenic region, were analyzed in 29 new-onset T1D children, and 30 age matched HCs using indirect ELISA. All eight peptides were highly recognized showing detectable reactivity. Results are summarized in Figure 1 and Figure 2.

MAP3865c_125–133_ Abs were detected in 34.5% of T1D subjects and in 6.7% of HCs, this difference was statistically significant (Fisher exact test: *p* = 0.0102; AUC = 0.72 Fig. 1A).

ZnT8_178–186_ Ab reactivity was very similar to that of its homologue MAP3865c_125–133_ when comparing T1D with HCs (34.5% and 6.7%, respectively; *p* = 0.0102; AUC = 0.75) (Fig. 1B).

MAP3865c_133–141_ Abs reactivity was the same obtained with the human homologue ZnT8_186–194_ when comparing T1D with HCs (55.2% and in 6.7% respectively; *p*<0.0001; AUC = 0.8 ) (Fig. 1C and Fig. 1D). MAP3865c_246–252_ Abs were detected in 27.6% of T1D subjects and in 3.3% of HCs, this difference being statistically significant (Fisher exact test: *p* = 0.01; AUC = 0.67) (Fig. 2A).

Ab positivity against MAP3865c_256–262_ was detected in 34.5% of T1D and only in 3.3% of HCs (*p* = 0.002; AUC = 0.63), showing a statistically significant higher frequency in T1D children (Fig. 2B).

MAP3865c_261–267_ Ab reactivity was identified in 31% of T1D subjects and in 3.3% of HCs, this difference being statistically significant (Fisher exact test: *p* = 0.005; AUC = 0.68) (Fig. 2C).

Ab positivity against the C-terminal MAP3865c_281–287_ was detected in 41.4% of T1D and only in 6.7% of HCs (*p* = 0.002; AUC = 0.7), showing a statistically significant higher frequency in T1D children (Fig. 2D).

**Figure 3:**
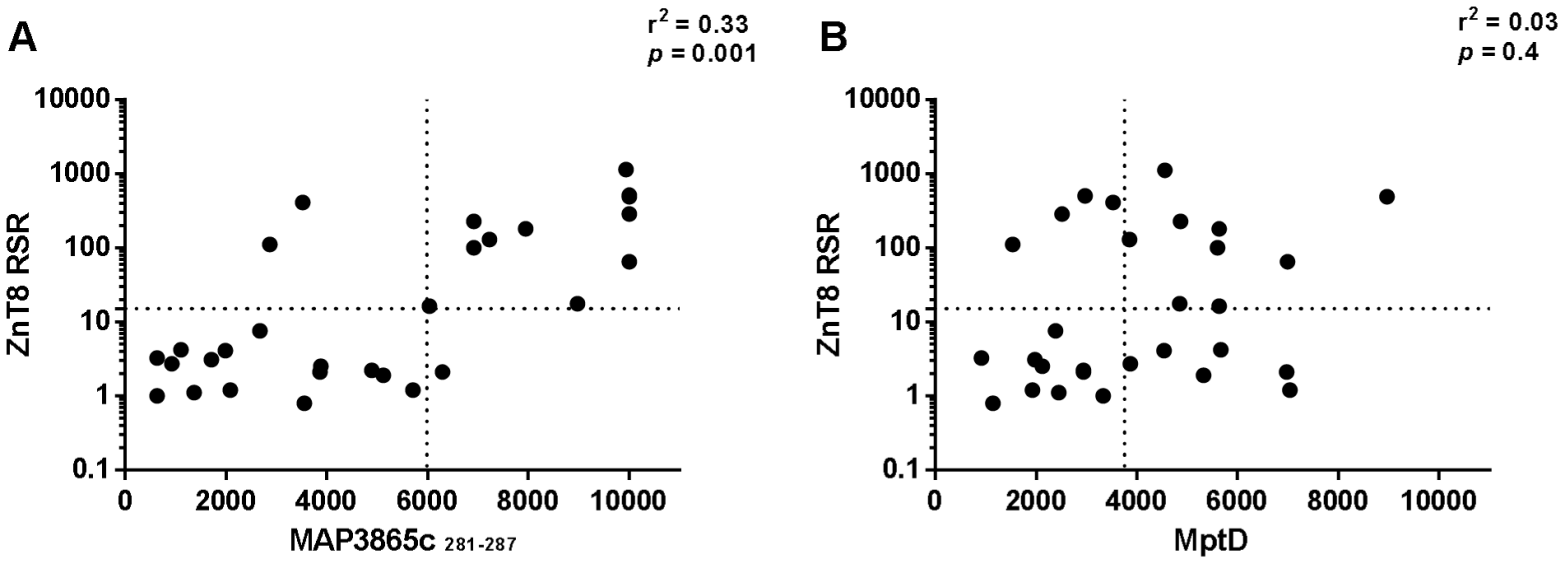
Correlation between titers of RSR ZnT8 Ab and MAP reactive Abs. Correlation is shown between titers of Abs recognizing (A) ZnT8RSR Abs and MAP3865c_281–287_ C-terminal epitope; (B) ZnT8RSR Abs and MptD MAP specific protein. Each circle represents the titer of one T1D child. The dotted line lines indicate the cut-off for positivity used in each assay, as calculated by ROC analysis.

The specificity of the MAP/Znt8 peptide based ELISA was further validated performing the RSR ZnT8 Ab ELISA test. Commercially available RSR ZnT8 Ab ELISA kit searches and identifies Abs against C-terminal residues 275–369 inclusive of the human ZnT8. After carrying out the RSR ZnT8 Ab ELISA 13 positives among 29 T1D (44.8%) were promptly identified, whereas none of the HCs was positive. The transmembrane MAP3865c/ZnT8 pairs of homologues peptides and the four C-terminal MAP3865c peptides were even more sensitive than the RSR ZnT8 Ab ELISA kit, in fact a range spanning from 8 to 16 (27.6–55.2%) out of 29 T1D patients, were correctly identified. Noteworthy, the best correlation between the RSR ZnT8 Ab ELISA kit and the C-terminal MAP3865c peptide is shown in Figure 3A.

Abs against the MAP-specific protein MptD were searched by indirect ELISA, highlighting that 14 out of 29 T1D children (48.3%) vs 1 out of 30 (3.3%) HCs were MptD Abs+ (*p*<0.0001). A correlation between Abs recognizing ZnT8RSR and Abs recognizing MptD MAP specific protein is shown in Fig. 3B.

Abs against GAD65, another major T1D autoantigen, and a totally T1D-unrelated protein ACHR (negative control) were also searched.

A similar percentage of Ab+ was found against GAD65 and MAP3865c peptides, whereas no blood sample of diabetic patients was reported to be positive for ACHR protein. Indeed, 12 out of 29 (41.4%) of T1D children and 2 out of 30 HCs (6.7%) were found to be positive after carrying out GAD65 ELISA testing.

If we look at T1D subjects, the high frequencies of Abs reacting against a MAP-specific protein MptD, together with the lack of Ab+ towards the T1D-unrelated protein ACHR, highlight the specificity of the MAP3865c/Znt8 based ELISA.

## Discussion

The objective of this study was to investigate whether ZnT8/MAP peptides were recognized in children at T1D-onset. We asked whether Abs against these epitopes may be markers of T1D in this pediatric population of Sardinia. This hypothesis is sustained by two major pieces of evidence.

Firstly, we have recently reported that MAP3865c and ZnT8 homologous sequences were cross-recognized by Abs in Sardinian T1D adults [6]. Secondly, it has been demonstrated that MAP infection triggers a specific humoral response in Sardinian T1D patients, which display high frequencies of Abs reacting against several mycobacterial proteins [10].

T1D increase is reported worldwide [11], and some evidence supports the role of infections as a cause of autoimmunity [12]. Our previous findings suggest that MAP infection is associated with T1D in the Sardinian population [6], [10]. Here, we report that Abs against MAP3865c_125–133_, MAP3865c_133–141_ and MAP3865c C terminal peptides in conjunction with ZnT8_178–186_ and ZnT8_186–194_ Abs are highly prevalent in Sardinian T1D children, but marginally so in HCs.

Interestingly, ZnT8-reactive CD8+ T cells are also predominantly directed against the ZnT8_186–194_ epitope and are detected in a majority of T1D patients outside Sardinia [13]. Indeed, Scotto et al. recently described that 60% of white HLA-A2(+) new T1D onset children, displayed ZnT8-reactive CD8(+) T cells capable of recognizing ZnT8_186–194_. A fraction ranging from 50% to 53% of the total ZnT8-specific CD8(+) T recognized (VAANIVLTV) epitope. The same epitope ZnT8_185–194,_ just one amino acid longer (AVAANIVLTV) was identified by Enee *et al*. along with other three Znt8 epitopes [14]. Of value, one of them ZnT8_291–300_ ILAVDGVLSV was highly conserved showing 50% aminoacid identity with the corresponding MAP region MAP3865c_228–236_ LGAVDGVTG. Another one was as well highly conserved showing 44% aminoacid identity with the homologue MAP region Znt8_228–236_ SISVLISAL and MAP3865c_167–174_ SLGVLIAG*(underlined aminoacid residues are conserved, * stands for gap).

It is known that the number of newly diagnosed cases positive for ZnT8 Abs is correlated with an older age at diagnosis of T1D in children and decline following diagnosis [3]. A trend towards an older age at T1D diagnosis was observed in children positive for Abs against Znt8_178–186_ (mean age at diagnosis 11.6±3.8 vs 7.6±3.9). Of note, titers against MAP3865c_133–141_ and ZnT8_186–194_ epitopes were similar to the one displayed by MAP3865c_125–133_, Znt8_178–186_ epitopes in T1D and healthy children. Indeed, Abs against MAP3865c_125–133_, Znt8_178–186_ had been previously found in 65.4% and 68% respectively, T1D adult from Sardinia. It is to establish if this is due to a loss of tolerance toward these epitopes in central lymphoid organs as well as in mature lymphocytes in the periphery. We here provide evidence for an association between Abs positivity for ZnT8 and MAP homologue epitopes in Sardinian T1D children. We were also able to demonstrate the specificity of the recognition, on account of the high frequencies of Abs reacting against MAP specific MptD protein, and the total lack of Ab+ towards ACHR (negative control) protein. The 2 T1D MptD negative patients (but positive for MAP3865c) may be explained by the lower sensitivity of the MptD ELISA (due to the poorly conserved conformation of the protein purified). Likewise, the C-terminal specificity of the MAP peptide based ELISA was confirmed by the RSR ZnT8 Ab ELISA results. Of note all children Abs+ for MAP3865c_133–141_ were also positive for its homolog ZnT8_186–194_ whereas only 7 out of 10 (70%) of children Abs+ for MAP3865c_125–133_ were as well positive for ZnT8_178–186._ This is a first step in understanding the possible role played by these epitopes in initiating autoimmunity. It is still early to conclude that these peptides are potential biomarker for disease prediction in T1D at risk children. Further work needs to analyze larger cohorts of children followed over time to elucidate whether Ab titers against these MAP/Znt8 epitopes also decrease after diagnosis. Moreover, it will be relevant to clarify whether MAP infection and seroreactivity appears before or after T1D onset. It would be also interesting to look for the presence of MAP in intestinal tissues of T1D patients [15]. This would be the first step to sort out among different interpretations: is MAP an innocent bystander that merely colonizes the host? Is its presence due to a secondary infection not involved in T1D etiology? Or is MAP a primary infectious agent triggering β-cell autoimmunity?

## Supporting Information

Table S1 Type 1 diabetes patients. *D: positive pediatric diabetes patient, n = 29; mean age 8.3±4.2 years; ^†^Days after diagnosis of T1D; ‡Positive if >15U/ml; § Positive if >4.45 ng/ml.
(DOC)
