# Supplementary material for: Zinc Transporter 8 and MAP3865c Homologous Epitopes are Recognized at T1D Onset in Sardinian Children
Source: PLoS One. 2013 May 17;8(5):e63371. doi: 10.1371/journal.pone.0063371 (PMC3656963; doi:10.1371/journal.pone.0063371)
Supplement: Table S1 — Type 1 diabetes patients. *D: positive pediatric diabetes patient, n = 29; mean age 8.3±4.2 years; †Days after diagnosis of T1D; ‡Positive if >15U/ml; § Positive if >4.45 ng/ml. (DOC) [file pone.0063371.s001.doc]

**Table S1.** Type 1 diabetes patients

| *Identity | Gender | Age | ^†^Days | ‡ZnT8 | § GAD65 |
| --- | --- | --- | --- | --- | --- |
| D.1 | M | 14 | 0 | 3.2 | 14.1 |
| D.2 | M | 13 | 0 | 2.1 | 20.4 |
| D.3 | F | - | - | 17.6 | 10.2 |
| D.4 | F | 14 | 0 | 178.7 | 5.2 |
| D.5 | M | 7 | 0 | 228.2 | 5.5 |
| D.6 | M | 8 | 0 | 1.2 | 1.7 |
| D.7 | M | 14 | 0 | 2.7 | 1 |
| D.8 | M | 8 | 0 | 3.1 | 1.8 |
| D.9 | M | 9 | 0 | 111.9 | 3.5 |
| D.10 | M | 4 | 24 | 2.1 | 6 |
| D.11 | F | 15 | 0 | 508.4 | 1.4 |
| D.12 | F | 5 | 25 | 1130.2 | 3 |
| D.13 | F | 12 | 0 | 1.1 | 1.3 |
| D.14 | F | 7 | 0 | 0.8 | 1.5 |
| D.15 | M | 2 | 20 | 2.5 | 1.7 |
| D.16 | M | 5 | 22 | 1 | 1.9 |
| D.17 | F | 4 | 2 | 16.3 | 1.9 |
| D.18 | F | 14 | 4 | 406.8 | 0.7 |
| D.19 | M | 14 | - | 129.9 | 4.6 |
| D.20 | M | 10 | 3 | 2.2 | 3.3 |
| D.21 | F | 5 | 1 | 1.9 | 2.4 |
| D.22 | F | 7 | 1 | 4.2 | 5.8 |
| D.23 | M | 9 | 3 | 99.5 | 5.4 |
| D.24 | F | 4 | 1 | 283.2 | 4.7 |
| D.25 | F | 4 | 2 | 1.2 | 11.5 |
| D.27 | M | 3 | 3 | 4.1 | 2.1 |
| D.28 | F | 13 | 3 | 7.5 | 1.2 |
| D.29 | F | 4 | 3 | 488.4 | 1.7 |
| D.30 | M | 5 | 1 | 65.1 | 5.1 |
